# Numerosity Comparison in Three Dimensions in the Case of Low Numerical Values

**DOI:** 10.3390/brainsci13060962

**Published:** 2023-06-17

**Authors:** Saori Aida

**Affiliations:** Graduate School of Sciences and Technology for Innovation, Yamaguchi University, 2-16-1 Tokiwadai, Ube 753-8611, Japan; saoaida@yamaguchi-u.ac.jp

**Keywords:** numerosity, number sense, stereopsis, depth, density, Weber’s law, visual perception, human cognition

## Abstract

This study investigated the perception of numbers in humans in 3D stimuli. Recent research has shown that number processing relies on “number sense” for small values, in line with Weber’s law. While previous studies have reported 3D numerosity overestimation mainly in higher numerical values, our experiment examined whether this phenomenon occurs at lower numerical values. We also explored whether the Weber ratio follows Weber’s law when comparing 2D and 3D stimuli in terms of the number of elements. Observers were presented with pairs of stimuli on a monitor and were asked to identify the stimulus with a larger number of elements. Using the constant method, we calculated the point of subjective equality (PSE), just noticeable difference (JND), and Weber ratios from the collected data. As a result, it was confirmed that the phenomenon of over-estimation of 3D numerical values occurs even when the numerical values are small. Additionally, we observed that the Weber fraction adhered to Weber’s law within the measured range. These findings contribute to the existing body of research, supporting the existence of distinct mechanisms for perceiving numerosity and density.

## 1. Introduction

In recent years, numerosity studies have demonstrated that animals, including humans, possess the ability to estimate the number of objects rapidly and reliably in their visual field, a phenomenon referred to as “number sense” [1]. Since infancy, humans possess the capacity to recognize the numerosity of objects present in their surroundings [2,3], and they use this ability every day. Humans can identify the number of objects in front of them with some accuracy. This ability is called number sense, and, since its proposal by [4], there have been many studies characterizing these subprocesses. Neurophysiological evidence from studies on monkeys [5], as well as neuroimaging results from studies on the human brain [6], have shown that this number sense relies on specialized brain mechanisms that are tuned to encode numerical information.

The direct or indirect nature of number perception, whether it arises from numerical or density cues, is a topic of debate among researchers [7,8,9,10,11]. The authors of [7] report numerosity adaptation. They found that after adapting to a group of dots with a higher number, the subsequent group of dots appeared to be fewer in number. This report showed that there is a numerosity mechanism. The authors of [9] argued that numerosity adaptation is a consequence of density adaptation. The authors of [12] found that the Weber ratio follows Weber’s law at low numerical values and decreases at high values. They argued that separate mechanisms exist for density and number estimates. For an area size of 8 and 14 arc degree in diameter, Weber’s law was followed when the number of elements was less than 15 and 30, and the square root law was followed when the number of elements was greater than 15 and 30, respectively. In other words, for an area size of 8 and 14 arc degree in diameter, the number of elements was about 15 and 30 when the number processing mechanism changed to a density processing mechanism, respectively. They called the case where the number of elements was less than the value when changing from the number processing mechanism to the density processing mechanism (15 and 30) a small number, and they called the case where the number of elements was greater than the value when changing from the number processing mechanism to the density processing mechanism (15 and 30) a large number.

Most studies on numerosity estimation have been presented on a 2D plane [13,14]. Previous studies have reported that the number of elements can be estimated almost accurately, even when the number of elements is relatively large [15,16]. It is reported that number judgments up to 100 with 2D stimuli are accurate in the constant method [15]. In addition, the accuracy and precision of number identification are influenced by several stimulus characteristics such as density, size of the element, luminance, size of the stimulus, retinal position, and element connectivity [7,17,18,19,20]. It has been reported that numerosity discrimination is also possible between different sensory modalities [15].

Recently, several studies on the numerosity (or density) presented in a 3D space have been reported [21,22,23,24,25]. The authors of [22] compared the number of elements of 3D stimuli with the number of elements of 2D stimuli and reported that the number of elements of 3D stimuli was perceived to be larger even when the number of elements presented was the same. This was called the 3D numerosity overestimation phenomenon. Earlier research indicated that the 3D numerosity overestimation phenomenon happens when the numerosity of stimulus elements falls within the range of 50 to 600. The number of elements used in the 3D numerosity overestimation phenomenon reported so far is larger than the number of elements with varying numerosity and density treatment mechanisms reported by [12]. The number of elements used in the previous experiments is within the range of the density processing mechanism, and it is possible that the 3D numerosity overestimation phenomenon was caused because of the density processing. They used the word “numerosity” in their experiments by having observers perform a numerosity discrimination task.

In this study, we will check whether the 3D numerosity overestimation phenomenon occurs with a number of elements in the range of 12 to 60 elements. In this study, the Weber fraction (thresholds normalized by perceived numerosity) for numerical discrimination is measured as a function of the number of elements. We will examine whether it obeys Weber’s law or the square root law. This analysis will clearly elucidate whether the 3D numerosity overestimation phenomenon is due to the number processing mechanism, the density processing mechanism, or both.

## 2. Materials and Methods

### 2.1. Experimental Devices

In the experiment, stimuli were generated using MATLAB on a Windows PC (LAVIE Direct DT PC-GD289ZZDL, NEC, Tokyo, Japan) and displayed on a monitor (CS230-CN, EIZO, Hakusan, Japan). The observer viewed the monitor with a stereoscope. The monitor has a resolution of 1920 by 1080 pixels. The viewing distance to the monitor was 60 cm for each experiment. The observer sat in a chair 42 cm above the floor in a dark room and fixed his/her head on a chin rest.

### 2.2. Stimuli

**Figure 1:**
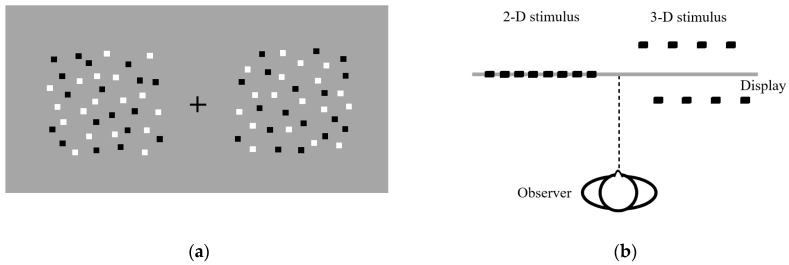
(**a**) Schematic front view of stimulus used in experiment. The width and the height of the 2D stimulus were the same as the 3D stimulus, respectively; (**b**) Schematic illustration of observer perception of appropriately fused stimuli. The observer perceives single surface and two surfaces, respectively.

The stimuli presented were in 2D and 3D formats using a random-dot stereogram (RDS). When fused, the 2D stimuli were perceived as a single surface, while the 3D stimuli appeared as two surfaces located at different depths but in the same visual direction [26] (see Figure 1a,b). The dimensions of the stimuli were 4.6 × 4.6 arc degrees and 6.1 × 6.1 arc degrees. A fixation cross was centered on the monitor and the disparity was adjusted to 0.0 relative to the monitor.

The stimuli were in 5.7 × 5.7 arcmin rectangles, displayed in white or black on a 27.1 × 46.2 arc degrees gray background. The luminance of the white elements, black elements, and gray background was measured using a luminance meter (LS100, Konica Minolta, Tokyo, Japan) and recorded as 3.10, 0.04, and 1.51 cd/m^2^, respectively. In the experiment, the 2D stimulus elements were presented with 0.0 arcmin disparity relative to the monitor surface, while the 3D stimulus elements had a disparity of 6.0 and 15.0 arcmin. Note that the magnitude of disparity used in this study is within the range where the 3D overestimation phenomenon occurs [21,22,23,24]. The disparity of the monitor surface was +3.0 arcmin for the front element and −3.0 arcmin for the back element for stimuli with a total disparity of 6.0 arcmin. For stimuli with a total disparity of 15.0 arcmin, the disparities were +7.5 arcmin and −7.5 arcmin, respectively. Positive signs indicate crossed disparity and negative signs indicate uncrossed disparity. The crossed disparity is perceived in front of the monitor surface, the uncrossed disparity is perceived behind the monitor surface, and the background is perceived behind the monitor surface. To prevent adjacent elements from overlapping or touching, the position of elements in all stimuli was randomly assigned and manipulated. Moreover, the position of 3D stimulus elements was adjusted to achieve binocular fusion [26].

In the experiment, the 2D stimuli were used as comparison stimuli, and the 3D stimuli were used as standard stimuli. The standard stimuli had the number of elements of 12, 24, 36, 48, and 60. When there were standard stimuli of 12, the comparison stimuli had a total number of elements of 6, 8, 10, 12, 14, 16, and 18. When there were standard stimuli of 24, the comparison stimuli had a total number of elements of 18, 20, 22, 24, 26, 28, and 30. When there were standard stimuli of 36, the comparison stimuli had a total number of elements of 24, 28, 32, 36, 40, 44, and 48. When there were standard stimuli of 48, the comparison stimuli had a total number of elements of 30, 36, 42, 48, 54, 60, and 66. When there were standard stimuli of 60, the comparison stimuli had a total number of elements of 36, 44, 52, 60, 68, 76, and 84. The 3D stimuli had two surfaces, with each surface having half of the total number of elements.

### 2.3. Observers

The sample sizes in our previously published study with a similar paradigm [22,23] were taken into consideration. We collected data from 7 observers in each experiment because the abovementioned study showed that this sample size yields ample power. The study included seven observers (one female, six males), aged between 21 and 36, with normal or corrected visual acuity and good stereopsis measured by the stereo fly test (Stereo Optical Company, Inc., Chicago, USA). All observers, except the author, provided informed consent and were not aware of the study’s goal. The experiment followed the ethical principles outlined in the Declaration of Helsinki and was approved by the Institutional Review Board of Yamaguchi University.

### 2.4. Procedure

For each trial in each experiment, observers were instructed to maintain their visual fixation and indicate which of the two stimuli, presented in juxtaposition on the monitor, exhibited a greater numerosity. Observers were instructed not to count. The stimuli were presented without temporal constraints, remaining visible until observers completed their response. The experimental design employed a within-subject approach. Observers were afforded the opportunity to take intermittent breaks following each block of trials to alleviate fatigue.

Observers underwent a stereopsis screening prior to each experimental session. They were instructed to verbally quantify the perceived depth in millimeters of a binocular disparity stimulus with two surfaces, for three distinct binocular disparities (4.0, 8.0, and 12.0 arcmin). Each stimulus was presented once, with a unique presentation order for each observer. Depth reports were plotted against disparity, and regression lines were calculated to determine the slope for each observer. Observers were deemed eligible for inclusion if their slope exceeded zero. The difficulty in separating the stereoscopic transparency stimulus into two surfaces, foreground and background, is when binocular disparity is small. Therefore, we considered that the perception of stereoscopic transparency would be possible if the two surfaces could be observed separately in all three conditions when viewed at 4.0, 8.0, and 12.0 arcmin, and if the depth increased as binocular disparity increased. The experiment encompassed practice and experimental sessions, with observers engaging in several training trials during the practice session until the experimenter deemed their comprehension of the task to be satisfactory.

The experiment encompassed four sessions, each featuring distinct combinations of binocular disparity and area size, as follows: (a) a combination of large area size and small binocular disparity; (b) a combination of small area size and small binocular disparity; (c) a combination of large area size and large binocular disparity; and (d) a combination of small area size and large binocular disparity. Within each session, there were five blocks, with the standard stimuli comprising 12, 24, 36, 48, and 60 elements. The presentation order of the five blocks varied among observers. In each block, the number of elements on the comparison stimulus and its presentation location were randomly chosen from seven different element quantities and two locations (right or left), respectively, with five repetitions. Consequently, each observer was exposed to stimuli a total of 1400 times (2 area sizes × 2 binocular disparities × 5 numbers of elements on the standard stimulus × 7 numbers of elements on the comparison stimulus × 2 locations × 5 repetitions).

### 2.5. Psychophysical Data Analysis

The study calculated PSE, JND, and Weber ratios from the experimental data. A psychometric function [22] was used to fit a plot of the percentage of trials where the number of elements in the 2D stimulus appeared greater than the number of elements in the 3D stimulus, against the number of elements in the 2D stimulus. PSE was the median value, JND was half the difference between the values at the 25th and 75th percentile points, and the Weber ratio was the JND divided by the PSE. Furthermore, we ascertained a bias in PSE by calculating the difference between PSE value and the number of elements in the standard stimulus. A positive bias indicates overestimation in numerical judgment, while a negative bias suggests underestimation. Subsequently, the calculated bias was divided by the number of elements on the standard stimulus, resulting in the bias of PSE ratio. A 10% overestimation relative to the number of elements of standard stimuli was predicted from previous studies [22]. Since the value of PSE is affected by the number of standard stimuli, the bias of PSE ratio was used to compare for the overestimation.

First, a three-way repeated measures ANOVA (5 number of elements × 2 binocular disparity × 2 aria size) on the difference was conducted. Next, the 95% confidence interval (95% CI) of the bias of the PSE ratio for each stimulus condition was analyzed, and if the 95% CI contained 0, the apparent values of the 2D and 3D stimuli were the same; if not, the apparent values of the 2D and 3D stimuli were different.

## 3. Results

First, a three-way repeated measures ANOVA (5 number of elements × 2 binocular disparity × 2 aria size) was conducted on the mean bias of the point of subjective equality (PSE) ratio. The analysis revealed that the main effect of the number of elements, *F*(4, 24) = 4.69, *p* = 0.0061, *η*^2^ = 0.085, was statistically significant, but the main effect of binocular disparity, *F*(1, 6) = 1.89, *p* = 0.22, *η*^2^ = 0.026; area size, *F*(1, 6) = 1.03, *p* = 0.35, *η*^2^ = 0.012; the three-way interaction effect, *F*(4, 24) = 0.03, *p* = 0. 99, *η*^2^ = 0.001; the simple interaction between binocular disparity and area size, *F*(1, 6) = 0.17, *p* = 0. 69, *η*^2^ = 0.001; the simple interaction between binocular disparity and the number of elements, *F*(4, 24) = 0.19, *p* = 0. 94, *η*^2^ = 0.003; and the simple interaction between area size and the number of elements, *F*(4, 24) = 0. 33, *p* = 0. 85, *η*^2^ = 0.003, were statistically insignificant.

**Figure 2:**
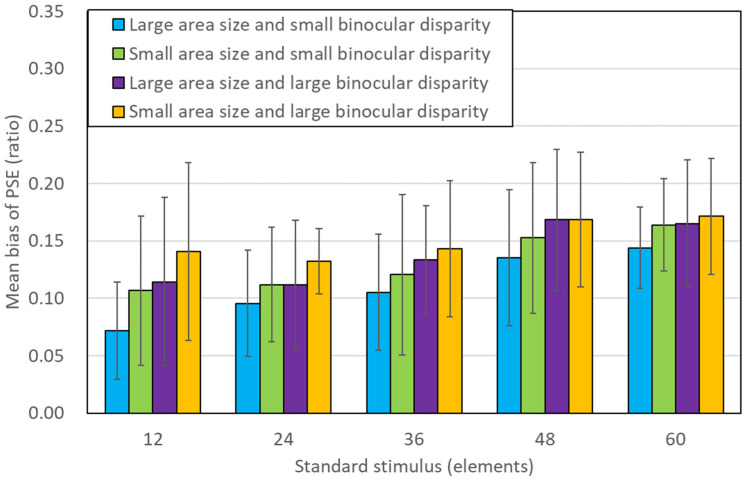
Results from experiment. Mean value of the PSE ratio for each number of elements in the standard stimulus. Each error bar represents a 95% CI.

Figure 2 shows the experimental results and mean value of the PSE ratio for each condition. The horizontal axis is the number of elements on the standard stimulus. The error bars in Figure 2 represent the 95% confidence intervals (95% CI). The mean PSE ratios in the four conditions are very similar, as illustrated in Figure 2, with 95% confidence intervals that overlap for each number of elements in the standard stimulus, in line with the ANOVA findings. 

Next, we assessed the 95% confidence intervals (95% CI) of the average PSE ratio for each condition to determine the 3D numerosity overestimation phenomenon. We considered an overestimation of the element count in the stereoscopic stimuli to have occurred when the lower limit was greater than 0. As seen in Figure 2, the lower limit was greater than 0, with 95% CIs (Table 1). These results suggest that 3D stimuli are perceived to contain more elements than 2D stimuli.

Third, just noticeable difference (JND) was analyzed using a three-way repeated measures ANOVA (5 number of elements × 2 binocular disparity × 2 aria size). The analysis revealed that the main effect of the number of elements, *F*(4, 24) = 54.72, *p* = 0.0000, *η*^2^ = 0.649, was statistically significant, but the main effect of binocular disparity, *F*(1, 6) = 0.30, *p* = 0.60, *η*^2^ = 0.001; area size, *F*(1, 6) = 0.01, *p* = 0.95, *η*^2^ = 0.000; the three-way cross effect, *F*(4, 24) = 1.22, *p* = 0.33, *η*^2^ = 0.012; the simple interaction between disparity and area size, *F*(1, 6) = 4.32, *p* = 0.08, *η*^2^ = 0.003; the simple interaction between disparity and the number of elements, *F*(4, 24) = 1.51, *p* = 0.23, *η*^2^ = 0.012; and the simple interaction between area size and the number of elements, *F*(4, 24) = 2.02, *p* = 0.12, *η*^2^ = 0.011, were statistically insignificant. 

**Figure 3:**
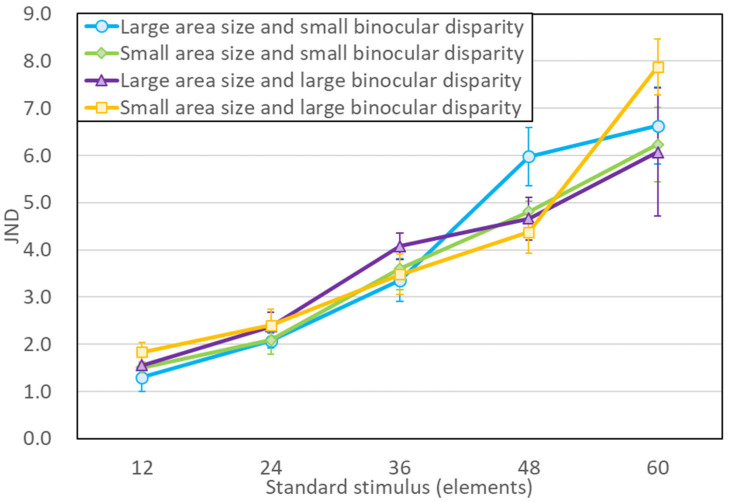
Results from experiment. Mean JND for each of the number of elements of the standard stimulus. Each error bar represents a 95% CI.

Figure 3 shows the experimental results and mean value of JND for each condition. The horizontal axis is the number of elements on the standard stimulus. The error bars indicate 95% CI. The similarity of the mean JNDs for the two binocular disparities and two area sizes, as illustrated in Figure 3, suggests that the task difficulty was consistent across stimulus conditions, which is supported by the ANOVA results.

Fourth, the Weber fraction was analyzed using a three-way repeated measures ANOVA (5 number of elements × 2 binocular disparity × 2 aria size). The results revealed a main effect of the number of elements, *F*(4, 24) = 2.67, *p* = 0.06, *η*^2^ = 0.093; a main effect of disparity, *F*(1, 6) = 1.15, *p* = 0.33, *η*^2^ = 0.005; a main effect of area size, *F*(1, 6) = 0.06, *p* = 0.82, *η*^2^ = 0.000; a three-way interaction effect, *F*(4, 24) = 0.84, *p* = 0. 51, *η*^2^ = 0.016; a binocular simple interaction effect between disparity and area size, *F*(1, 6) = 0.46, *p* = 0.52, *η*^2^ = 0.002; a simple interaction effect between binocular disparity and the number of elements, *F*(4, 24) = 1.89, *p* = 0.15, *η*^2^ = 0.028; and a simple interaction effect between area size and the number of elements, *F*(4, 24) = 1.62, *p* = 0.20, *η*^2^ = 0.022, were statistically insignificant. 

**Figure 4:**
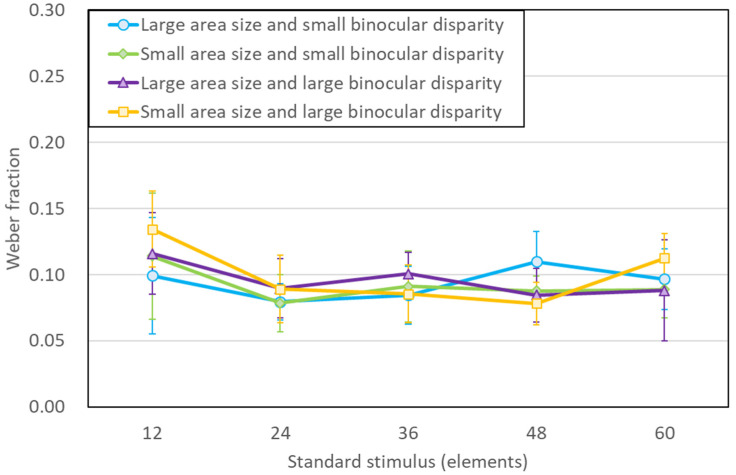
Results from experiment. Mean Weber fraction for each of the number of elements of the standard stimulus. Each error bar represents a 95% CI.

Figure 4 shows the experimental results and mean value of the Weber fraction for each condition. The horizontal axis is the number of elements on the standard stimulus. The error bars indicate 95% CI. The ANOVA findings agree with the mean Weber fractions illustrated in Figure 4, demonstrating that the task complexity was comparable among the stimulus conditions for both binocular disparities and area sizes.

## 4. Discussion

The purpose of the experiment conducted in this study was to verify if the overestimation of 3D numerosity occurs at low numerical values. The results of the experiment confirmed that the 3D numerosity overestimation phenomenon occurs under all conditions in which this experiment was conducted. Binocular disparity, range of presentation, and density had no effect on the phenomenon in the range covered by this experiment. The experiment also investigated the Weber fraction. The experimental results showed that the Weber fraction was a constant value over the range of conditions treated in this experiment.

Previous experiments dealing with the 3D numerosity overestimation phenomenon dealt with a number of elements of 50 to 600. In the present experiment, we used the number of elements of stimuli, 12 to 60, to see if the phenomenon occurred. The results of the experiment indicated that the phenomenon was present across all the conditions under which the study was carried out. This suggests that the 3D numerosity overestimation phenomenon occurs over a wide range of numerosities.

In this study, task difficulty was also compared using the JND and Weber fractions as indices. According to the experimental findings, when the element count is constant, the level of task difficulty and the sensitivity to discrimination are similar for both 2D and 3D stimuli, depending on the stimulus conditions. This result is also consistent with that of [23]. Thus, the 3D of the elements affects number perception but not number sensitivity.

In many previous studies, number sense by humans and other animals follows Weber’s law [27]. The authors of [12] found that the Weber ratio follows Weber’s law at low numerical values and decreases at high values. For the range of the number of elements conducted in this experiment, the Weber law could be followed as in [12]. The implication of this result is that the mechanism for estimating numbers is also employed to determine the number of elements in the 3D stimulus. In Experiment 2 of [22], the Weber fractions were 0.10 (SE 0.043) and 0.05 (SE 0.01) in the 150 and 300 elements conditions, respectively. Nonetheless, their experiments differed in the luminance conditions, presentation range, and presentation method used. The results indicated that Weber fractions were smaller at high values compared to those at low numerical values. These results indicate that the 3D numerosity overestimation phenomenon is due to both the number-processing mechanism and the density-processing mechanism.

The dot densities of the stimuli used in this study ranged from 0.33 to 2.89 dots/degree^2^, which are higher than those of [12]. However, if considered in terms of the dot density of a rectangular volume, the 3D stimuli would be perceived as sparser. The current study and [22] show that the Weber fractions decreased with an increase in the number of elements. The present study compared 3D stimuli to 2D stimuli at low numerosities and observed the 3D numerosity overestimation phenomenon previously seen in other studies. The Weber fractions followed Weber’s law in the measurement range of this experiment, thus reinforcing and broadening earlier studies that demonstrate separate mechanisms for perceived numerosity and density.

One potential mechanism that may lead to the 3D numerosity overestimation phenomenon is the interference caused by processing the binocular disparity of the stimulus elements [23]. Binocular disparity refers to the differences in the images projected onto the two retinas, allowing us to perceive depth in a scene. It was proposed that when processing the disparities among the elements in a 3D stimulus, there is interference with the accurate estimation of numerosity [23]. This interference may arise due to the complex computations required to integrate the depth of information with the perception of numerosity. As a result, the perceived numerosity may be inflated, leading to the overestimation of the number of elements in the 3D stimulus. Furthermore, the depth structure of the stimulus plays a crucial role in the 3D numerosity overestimation phenomenon [23,24,25]. It has been found that the background is overestimated when observing a motion stereoscopic transparency stimulus with two surfaces [25]. The overlapping of stereo surfaces in depth has been found to enhance the magnitude of overestimation compared to non-overlapping surfaces [23]. This suggests that the spatial arrangement and organization of the elements in 3D scenes interact with the processing of binocular disparity, influencing the extent of overestimation.

In conclusion, our experiment confirmed the occurrence of 3D numerosity overestimation at low numerical values, and our results indicate that this phenomenon persists across different conditions, including variations in binocular disparity, range of presentation, and density. The precision of numerosity perception, as reflected by the Weber fraction, remained constant across the range of conditions tested. These findings contribute to our understanding of numerosity perception in 3D scenes and have important implications for clinicians, neuroscientists, and psychologists studying numerosity perception in real-world contexts. Further research is needed to investigate the potential influence of other contextual factors and to replicate and extend our findings.

## Figures and Tables

**Table 1:** Mean bias of PSE ratio and 95% CI.

Condition	12 Elements		24 Elements		36 Elements		48 Elements		60 Elements	
	Mean Bias of PSE Ratio	95% CI	Mean Bias of PSE Ratio	95% CI	Mean Bias of PSE Ratio	95% CI	Mean Bias of PSE Ratio	95% CI	Mean Bias of PSE Ratio	95% CI
Large area size and small binocular disparity	0.07	±0.04	0.1	±0.05	0.11	±0.05	0.14	±0.06	0.14	±0.04
Small area size and small binocular disparity	0.11	±0.07	0.11	±0.05	0.12	±0.07	0.15	±0.07	0.16	±0.04
Large area size and large binocular disparity	0.11	±0.07	0.11	±0.06	0.13	±0.05	0.17	±0.06	0.17	±0.06
Small area size and large binocular disparity	0.14	±0.08	0.13	±0.03	0.14	±0.06	0.17	±0.06	0.17	±0.05

## Data Availability

The data and materials for all experiments will be available upon request.
